# Therapeutic Potential of Natural Compounds for Brain Ischemia-Reperfusion Injury

**DOI:** 10.3390/biology14091153

**Published:** 2025-09-01

**Authors:** Ki-Yeon Yoo, Moo-Ho Won, Ji Hyeon Ahn, Joon Ha Park

**Affiliations:** 1 Department of Anatomy, College of Dentistry, Gangneung-Wonju National University, Gangneung 25457, Republic of Korea; kyyoo@gwnu.ac.kr; 2 Research Institute for Dental Engineering, Gangneung-Wonju National University, Gangneung 25457, Republic of Korea; 3 Department of Emergency Medicine, School of Medicine, Kangwon National University, Chuncheon 24341, Republic of Korea; mhwon@kangwon.ac.kr; 4 Department of Physical Therapy, College of Health Science, Youngsan University, Yangsan 50510, Republic of Korea; jh-ahn@ysu.ac.kr; 5 Department of Anatomy, College of Korean Medicine, Dongguk University, Gyeongju 38066, Republic of Korea

**Keywords:** ischemic stroke, reperfusion injury, neuroprotection, antioxidant therapy, blood-brain barrier, neuroinflammation, natural product

## Abstract

Brain ischemia-reperfusion (I/R) injury, commonly occurring in ischemic stroke and post-cardiac arrest scenarios, results in complex secondary damage involving oxidative stress, inflammation, apoptosis, and blood-brain barrier (BBB) breakdown. Despite decades of research, no pharmacological agent has yet been clinically approved for post-I/R neuroprotection. Natural compounds have recently gained attention for their multimodal therapeutic potential, including antioxidant, anti-inflammatory, anti-apoptotic, and neuroregenerative effects. This review highlights nine promising candidates—resveratrol, curcumin, quercetin, berberine, ginkgolide B, baicalin, naringin, fucoidan, and astaxanthin—that exhibit efficacy in experimental models of I/R injury when administered after the insult. Their chemical structures, pharmacokinetics, and mechanisms of action are described in detail, focusing on key signaling pathways such as nuclear factor erythroid 2-related (Nrf2), nuclear factor kappa B (NF-*κ*B), phosphatidylinositol 3-kinase/protein kinase B (PI3K/Akt), and brain-derived neurotrophic factor (BDNF). Importantly, we outline the selection criteria for these compounds, including demonstrated neuroprotective efficacy, mechanistic clarity, and translational feasibility. While several challenges remain—such as limited bioavailability, BBB penetration, and species-specific metabolism—emerging strategies like nanoparticle delivery, synthetic analogs, and drug combinations offer potential solutions. By emphasizing the therapeutic versatility and mechanistic diversity of these natural agents, this review supports their clinical potential and encourages further preclinical optimization and biomarker-guided human trials.

## Simple Summary

(1) Nine natural compounds show therapeutic effects after brain I/R injury onset. (2) Compounds target oxidative stress, inflammation, apoptosis, BBB, and neurogenesis. (3) Key signaling pathways include Nrf2, NF-*κ*B, PI3K/Akt, and BDNF. (4) Challenges include low bioavailability and poor BBB penetration. (5) Nanocarriers and synthetic derivatives improve delivery and translation potential.

## 1. Introduction

Brain ischemic insults refer to conditions where blood flow to the brain is restricted or completely blocked, leading to oxygen and nutrient deprivation. Brain ischemic insults include ischemic stroke, transient ischemic attack (TIA), and ischemia-reperfusion (I/R) injury [1,2,3]. Ischemic stroke is a permanent blockage of a cerebral artery, leading to prolonged ischemia and subsequent neuronal damage; TIA is a temporary episode of cerebral ischemia with no lasting damage [3]. In contrast, I/R injury arises not only from the initial ischemic episode but also from the subsequent restoration of blood flow, which paradoxically aggravates brain injury through multiple secondary cascades [2]. Table 1 outlines the key distinctions and commonalities among these conditions.

**Figure 1:**
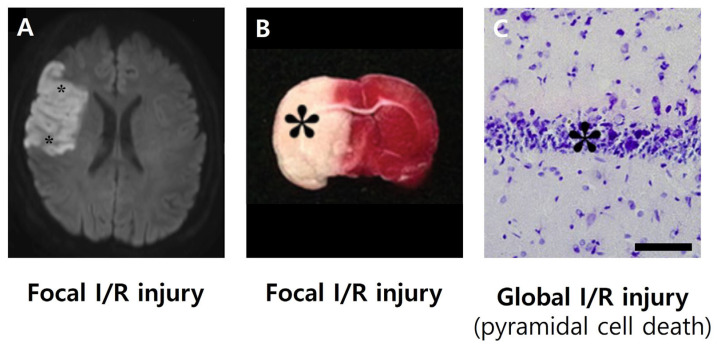
Representative imaging of brain I/R injury in humans and animal models. (**A**): Diffusion-weighted MRI (DWI) from a human patient with focal cerebral I/R injury reveals hyperintense regions (asterisks) indicating acute infarction in the middle cerebral artery territory [9]. (**B**): 2,3,5-triphenyltetrazolium chloride (TTC)-stained coronal brain sections from a rat subjected to MCAO, a model of focal I/R injury [10]. White regions (asterisk) indicate infarcted tissue, while red areas represent viable brain. (**C**): Cresyl violet-stained hippocampal sections from a gerbil following global cerebral I/R injury show significant neuronal loss (asterisk) in the CA1 region, a hallmark of delayed pyramidal cell death [11].

As illustrated in Figure 1, cerebral I/R injury triggers a complex pathophysiological process following reperfusion, including oxidative stress, excitotoxicity, neuroinflammation, mitochondrial dysfunction, and apoptosis [4,5,6]. Despite extensive research efforts, no neuroprotective agents have been approved for clinical use in the post-ischemic phase. This therapeutic gap has spurred interest in bioactive natural compounds with multimodal protective properties [7,8].

**Table 1:** Key differences and similarities among ischemic stroke, TIA, and I/R injury.

Feature	Ischemic Stroke	TIA	I/R Injury	Representative References
Duration	>24 h	<24 h	Variable (depends on insult)	[1,2]
Recovery	Often incomplete (permanent damage)	Complete recovery	Variable, but often worsens with reperfusion	[3,4]
Pathological Outcome	Infarction, necrosis	No infarction	Infarction, oxidative stress, BBB disruption	[2,4,5]
Main Cause	Thrombosis, embolism	Thrombosis, embolism (transient)	Reperfusion after ischemia	[2,3]
Treatment	Thrombolysis, thrombectomy	Risk reduction (e.g., anticoagulants)	Antioxidants, anti-inflammatory therapies	[6,7,8]

Natural compounds possess broad pharmacological effects, including antioxidant, anti-inflammatory, anti-apoptotic, and neuroregenerative actions. While many compounds have demonstrated neuroprotection in pre-treatment paradigms, therapeutic neuroprotection—intervention after I/R onset—is particularly relevant for clinical translation [12]. Preventive strategies typically rely on ischemic preconditioning and mitochondrial priming prior to injury, which are not feasible in unpredictable events such as stroke. By contrast, post-injury interventions must operate under narrow therapeutic windows, target rapidly evolving secondary injury cascades, and overcome blood-brain barrier (BBB) disruption during acute reperfusion [4,7].

Given these challenges, compounds with proven efficacy in post-treatment models hold greater translational promise. Natural product–based drugs exhibit greater structural diversity and stereochemical complexity than purely synthetic drugs, which contributes to enhanced chemical diversity and potentially lower toxicity in drug development [13]. In this review, we focus on nine natural compounds with demonstrated post-I/R efficacy in preclinical studies: resveratrol, curcumin, quercetin, berberine, ginkgolide B, baicalin, naringin, fucoidan, and astaxanthin. Their chemical structures, molecular mechanisms, and post-injury neuroprotective outcomes are summarized and compared.

In this review, nine natural compounds were selected based on the following criteria: (1) published experimental evidence supporting therapeutic efficacy when administered after brain I/R injury (i.e., post-treatment); (2) representation of diverse chemical classes including polyphenols, alkaloids, flavonoids, carotenoids, terpenoids, and polysaccharides; and (3) evidence of molecular mechanisms related to neuroprotection, such as antioxidant, anti-inflammatory, and anti-apoptotic pathways. Additionally, compounds with potential translational value based on preclinical models or preliminary clinical data were prioritized.

## 2. Pathophysiology of Brain I/R Injury

**Figure 2:**
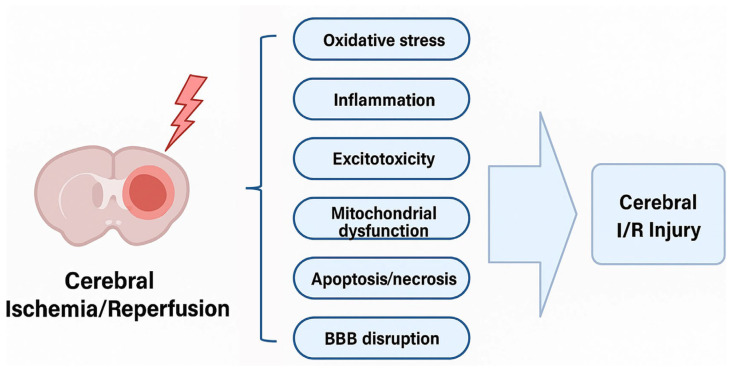
Pathophysiological mechanisms underlying brain I/R injury. The I/R event initiates a cascade of interrelated cellular and molecular responses, including oxidative stress, neuroinflammation, excitotoxicity, mitochondrial dysfunction, apoptosis/necrosis, and BBB disruption. These pathological processes interact to promote neuronal death and neurological deficits. Understanding these mechanisms is essential for identifying therapeutic targets and developing interventions to mitigate I/R-induced brain damage.

Brain I/R injury results from the restoration of blood flow to previously ischemic brain tissue, which paradoxically exacerbates neuronal injury. Table 2 and Figure 2 show pathophysiological mechanisms of brain I/R injury, but the mechanisms are complex, including oxidative stress, inflammation, excitotoxicity, mitochondrial dysfunction, apoptosis/necrosis, and BBB disruption [2,4,14].

### 2.1. Oxidative Stress

The sudden influx of oxygen during reperfusion following cerebral ischemia results in a burst of reactive oxygen species (ROS) production, including superoxide anion, hydrogen peroxide, and hydroxyl radicals. These free radicals attack cellular components, leading to lipid peroxidation, protein oxidation, and DNA damage, ultimately disrupting cellular homeostasis and viability. Such oxidative stress is a primary contributor to neuronal death and secondary brain injury [6,15]. Nuclear factor erythroid 2–related factor 2 (Nrf2) is a transcription factor that regulates the expression of antioxidant enzymes, including heme oxygenase-1 (HO-1), superoxide dismutase (SOD), and catalase. Activation of the Nrf2/ARE (antioxidant response element) pathway has been shown to attenuate ROS-induced injury after I/R [6]. In addition, ferroptosis, a form of regulated necrosis triggered by iron overload and lipid peroxidation, is increasingly recognized in I/R-induced neuronal death. Nrf2 signaling alleviates ferroptotic damage by regulating iron metabolism and lipid ROS detoxification [15].

In a rat model of focal cerebral ischemia induced by middle cerebral artery occlusion (MCAO) [16,17,18], oxidative stress is evidenced by increased malondialdehyde (MDA, a marker of lipid peroxidation) and decreased SOD levels in brain tissues. Treatment with the antioxidant astaxanthin significantly reverses these changes and reduces infarct volume and neurological deficits in a dose-dependent manner. Similarly, in global cerebral I/R models, such as the bilateral common carotid artery occlusion (BCCAO) [19,20,21,22] or four-vessel occlusion (4VO) models [23,24,25], increased levels of ROS and oxidative biomarkers have been observed, particularly in hippocampal regions vulnerable to delayed neuronal death. These models also show depletion of endogenous antioxidant systems. Experimental antioxidant treatments in global models demonstrate protective effects similar to those observed in focal models, supporting the centrality of oxidative stress in I/R pathology across different ischemia paradigms.

### 2.2. Inflammation

Following cerebral I/R injury, a robust inflammatory response is rapidly initiated. Activated neuroglial cells, especially microglia and astrocytes, play a central role in this process by releasing a cascade of pro-inflammatory cytokines such as tumor necrosis factor-alpha (TNF-*α*), interleukin-1 beta (IL-1*β*), and IL-6. These cytokines not only directly contribute to neuronal death but also exacerbate secondary injury through promotion of leukocyte infiltration, disruption of the BBB, and amplification of oxidative stress [5,7,8]. In particular, microglial activation is a hallmark of neuroinflammation, and its polarization toward a pro-inflammatory M1 phenotype is associated with the upregulation of inducible nitric oxide synthase (iNOS), cyclooxygenase-2 (COX-2), and cytokines [12,26]. Astrocytes, in response to ischemia, undergo reactive gliosis and contribute to the neuroinflammatory milieu through the secretion of inflammatory mediators and alterations in glutamate homeostasis [27,28].

In focal cerebral I/R models using MCAO, elevated levels of TNF-*α* and IL-1*β* in peri-infarct tissue are well documented. For instance, in an MCAO rat model, curcumin administration significantly reduces microglial activation and downregulates the expression of TNF-*α* and IL-1*β*, demonstrating potent anti-inflammatory activity [29]. Similarly, resveratrol and baicalin were shown to suppress nuclear factor kappa B (NF-*κ*B) signaling, a key transcription factor that drives pro-inflammatory gene expression after I/R [12]. In global I/R models, such as BCCAO and 4VO, microglial activation and cytokine expression in the hippocampus peak within 24 h post-reperfusion and persist during the delayed neuronal death phase [27,30,31]. Anti-inflammatory treatment in these models, including berberine, results in attenuation of microglial reactivity and preservation of hippocampal neurons [32]. These findings reinforce the pivotal role of inflammation in mediating damage after both focal and global cerebral I/R injuries.

### 2.3. Excitotoxicity

Excitotoxicity is a key contributor to neuronal injury in the aftermath of cerebral I/R. It is primarily driven by the pathological accumulation of extracellular glutamate, a major excitatory neurotransmitter. During ischemia, the depletion of adenosine triphosphate (ATP) impairs the function of ion pumps and glutamate transporters, particularly in astrocytes and neurons. This dysfunction leads to an unchecked release and reduced reuptake of glutamate, which overstimulates glutamate receptors—especially N-methyl-D-aspartate (NMDA) and *α*-amino-3-hydroxy-5-methyl-4-isoxazolepropionic acid (AMPA) receptors—on postsynaptic neurons [1,33]. The overactivation of NMDA receptors results in excessive influx of calcium ions (Ca^2+^), activating a cascade of intracellular signaling events including calpains, nitric oxide synthase (NOS), and phospholipases, which collectively trigger oxidative stress, mitochondrial dysfunction, and ultimately, neuronal apoptosis or necrosis [33,34].

In vitro studies using PC12 cells and primary neurons exposed to glutamate demonstrate calcium overload, ROS accumulation, and cell death, which can be attenuated by agents such as curcumin or memantine that modulate glutamate receptor signaling [34]. In vivo, focal ischemia models like MCAO show elevated extracellular glutamate levels within minutes of ischemia onset, persisting during early reperfusion. Treatment with NMDA receptor antagonists or glutamate release inhibitors significantly reduces infarct volume and improves neurological outcomes. For example, riluzole (a glutamate release inhibitor) and postsynaptic density-95 inhibitors have shown neuroprotective effects by limiting excitotoxic signaling [35,36,37]. Global I/R models such as 4VO in rats also display significant glutamate elevation in the hippocampus and cortex, regions highly susceptible to delayed neuronal death. Excitotoxicity in these models is associated with synaptic remodeling deficits and long-term cognitive impairment. Interventions aimed at modulating glutamatergic signaling in these global models—e.g., treatment with magnesium sulfate or NMDA receptor blockers—ameliorate histopathological and behavioral outcomes [38,39].

### 2.4. Mitochondrial Dysfunction

Mitochondria are central organelles in determining neuronal fate during I/R injury [40]. Ischemia impairs oxidative phosphorylation and ATP production, while reperfusion paradoxically causes a surge of ROS, calcium overload, and mitochondrial membrane damage. One of the pivotal events in mitochondrial-mediated neuronal injury is the opening of the mitochondrial permeability transition pore (mPTP), which disrupts the mitochondrial membrane potential (Δψm), impairs ATP synthesis, and promotes cytochrome c release into the cytoplasm, leading to caspase-3 activation and apoptosis [2,41].

In focal I/R models, such as MCAO, ultrastructural analyses reveal mitochondrial swelling, cristae fragmentation, and bioenergetic failure. Pharmacological agents targeting mitochondrial preservation—such as cyclosporine A (mPTP inhibitor)—ameliorate neuronal damage and reduce infarct size [42,43]. In global I/R models like two- or four-vessel occlusion, similar mitochondrial pathology is observed in vulnerable regions such as the hippocampal CA1, with delayed neuronal death linked to mitochondrial oxidative damage and dysregulated calcium homeostasis, and, in the models, cyclosporine A or MitoQ (a mitochondria-targeted antioxidant) ameliorates or protects neuronal damage in the hippocampus [44,45,46].

Recent studies emphasize the importance of maintaining mitochondrial dynamics, particularly the balance between fission and fusion, for neuronal survival [47]. Excessive mitochondrial fission, often mediated by the GTPase Drp1 (dynamin-related protein 1), leads to mitochondrial fragmentation and apoptosis. Conversely, promotion of fusion proteins like Mfn1/2 or OPA1 supports mitochondrial integrity. Furthermore, mitophagy—a selective form of autophagy that removes damaged mitochondria—is neuroprotective if appropriately regulated but can lead to energy depletion if excessive [47,48]. Targeting mitochondrial biogenesis, dynamics, and quality control through pharmacological agents or natural compounds (e.g., resveratrol, berberine) holds promise in minimizing mitochondrial dysfunction in both focal and global I/R injuries.

### 2.5. Apoptosis and Necrosis

Programmed cell death mechanisms—particularly apoptosis—play a central role in neuronal loss after cerebral I/R injury. Apoptosis is mediated by intrinsic (mitochondrial) and extrinsic (death receptor) pathways. The intrinsic pathway is initiated by mitochondrial outer membrane permeabilization, often in response to oxidative stress, calcium dysregulation, or DNA damage. This results in cytochrome c release, apoptosome formation, and activation of caspase-9, followed by downstream caspase-3, ultimately leading to DNA fragmentation and cell dismantling. The extrinsic pathway involves ligand binding to death receptors such as Fas or TNF receptor, which directly activates caspase-8 and downstream effectors [5,49,50]. Key molecular regulators of apoptosis include members of the B-cell leukemia/lymphoma 2 (Bcl-2) protein family. Pro-apoptotic proteins like Bcl-2-associated X protein (Bax) and Bcl-2 antagonist killer 1 (Bak) promote mitochondrial permeabilization, while anti-apoptotic proteins such as Bcl-2 and Bcl-xL inhibit this process. A shift in the Bcl-2/Bax ratio toward pro-apoptotic signaling is consistently observed in I/R models, indicating the activation of apoptotic machinery. In parallel, severe or prolonged ischemic insult leads to necrosis—an uncontrolled form of cell death characterized by cellular swelling, membrane rupture, and inflammatory cell infiltration [49,50,51].

In focal ischemia models using MCAO, increased expression of cleaved caspase-3, TdT-mediated dUTP nick end labeling (TUNEL)-positive cells, and Bcl-2 downregulation is noted in peri-infarct zones. Treatments with natural compounds such as curcumin restore the Bcl-2/Bax balance, inhibit caspase activation, and improve functional recovery [52,53]. Resveratrol has also been shown to upregulate silent information regulator of transcription1 (SIRT1) signaling, which exerts anti-apoptotic effects via modulation of p53 and forkhead box O (FOXO) transcription factors [51]. In global cerebral I/R models (e.g., 4VO and BCCAO), apoptosis is prominently observed in hippocampal CA1 pyramidal neurons, where delayed neuronal death occurs within 48–72 h after reperfusion. Apoptotic markers, including cytochrome c release and active caspase-3, colocalize with neuronal damage in these regions [53]. Experimental treatments targeting apoptotic pathways reduce hippocampal neurodegeneration and improve cognitive outcomes [54,55,56].

### 2.6. BBB Disruption

The BBB is a highly selective semipermeable interface formed by endothelial cells, pericytes, astrocytic endfeet, and the basal lamina. It regulates the passage of ions, nutrients, and cells between the bloodstream and the central nervous system, maintaining cerebral homeostasis [57,58]. Cerebral I/R injury disrupts the BBB structure and function primarily through oxidative stress, inflammation, and protease activation, which contribute to tight junction degradation and increased permeability [4,59]. During ischemia, decreased ATP levels compromise endothelial cell integrity, while reperfusion exacerbates oxidative damage via excessive generation of ROS and inflammatory cytokines. These events lead to downregulation and disassembly of tight junction proteins such as claudin-5, occludin, tricellulin, and zonula occludens-1 [60,61]. Matrix metalloproteinases (MMPs), especially MMP-2 and MMP-9, are upregulated during I/R and cleave extracellular matrix components and tight junction proteins, worsening BBB breakdown and allowing peripheral immune cell infiltration [59,60]. In addition, it is suggested that astrocytic endfeet and pericyte function are crucial to BBB maintenance during and after ischemia. Disruption of aquaporin-4 polarization and endothelial-pericyte communication contributes to capillary leakage and microvascular collapse, amplifying secondary brain injury [62,63]. Therapeutic strategies aimed at stabilizing endothelial-astrocyte interactions and inhibiting MMPs are thus emerging as promising interventions to preserve BBB function.

In focal I/R models using MCAO, Evans blue extravasation studies, and immunohistochemistry demonstrate leakage across the BBB and reduced claudin-5 expression in ischemic areas [64,65]. Experimental treatment with fucoidan restores tight junction protein expression and reduces vasogenic edema and leukocyte infiltration, highlighting its protective role [66]. In addition, baicalin reduces I/R-induced neuronal damage, brain edema, and BBB permeability, which might be related to inhibition of MMP-9 expression and MMP-9-mediated occludin degradation [67]. In global cerebral I/R models, BBB disruption is prominent in the hippocampus, cortex, and thalamus. Evidence includes leakage of serum proteins (e.g., immunoglobulin G (IgG), albumin), increased water content (edema), and decreased expression of junctional proteins [68,69]. In addition, decursin has been shown to protect hippocampal neurons from I/R injury, to attenuate I/R-induced BBB leakage and damage of astrocyte endfeet [70].

**Table 2:** Pathophysiological Mechanisms of Brain I/R Injury.

Pathophysiological Mechanism	Key Features	Representative References
Oxidative stress	ROS overproduction, lipid peroxidation, DNA/protein damage; Nrf2/ARE activation counters injury	[6,15,16,17,18,19,20,21,22,23,24,25]
Inflammation	Cytokine release (TNF-*α*, IL-1*β*), microglial activation, NF-*κ*B pathway, astrocyte gliosis	[5,7,8,12,26,27,28,29,30,31,32]
Excitotoxicity	Glutamate accumulation, NMDA/AMPA overactivation, Ca^2+^ influx, oxidative stress, synaptic loss	[1,33,34,35,36,37,38,39]
Mitochondrial dysfunction	ATP depletion, mPTP opening, cytochrome c release, Drp1-mediated fission, impaired mitophagy	[2,40,41,42,43,44,45,46,47,48]
Apoptosis and necrosis	Caspase cascade (3, 8, 9), Bcl-2/Bax ratio shift, intrinsic/extrinsic apoptosis, SIRT1 signaling	[5,49,50,51,52,53,54,55,56]
BBB disruption	Tight junction protein loss, MMP-9 upregulation, astrocyte endfeet detachment, IgG/albumin leakage	[4,57,58,59,60,61,62,63,64,65,66,67,68,69,70]

## 3. Natural Compounds with Therapeutic Potential for Brain I/R Injury

**Figure 3:**
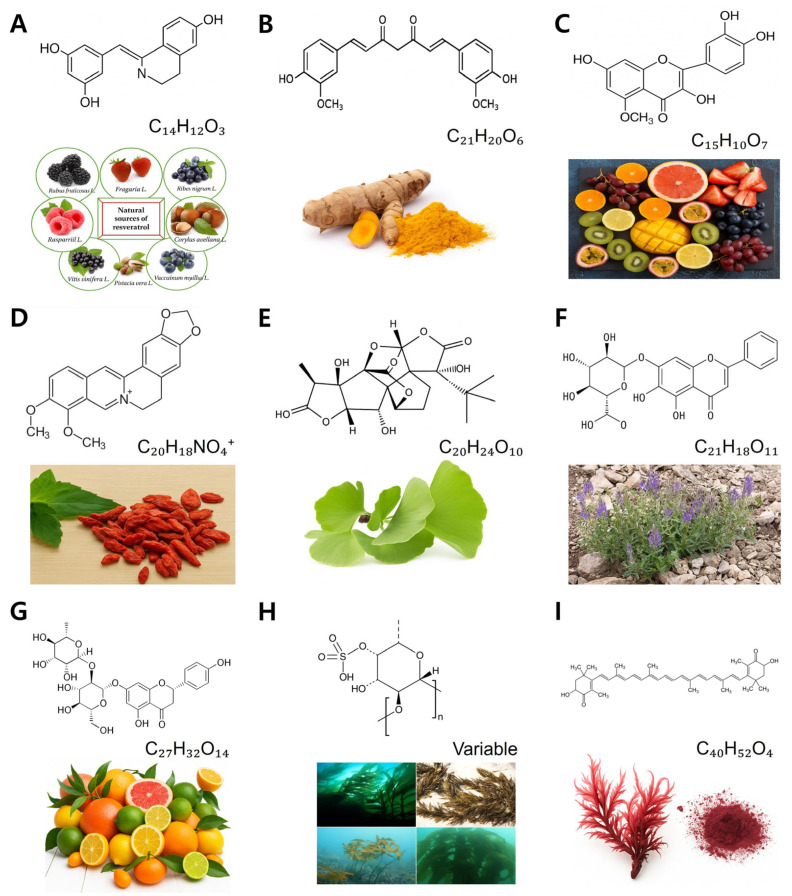
Chemical structures of nine natural compounds with demonstrated post-I/R therapeutic effects. (**A**–**C**): resveratrol, curcumin, quercetin. (**D**–**F**): row: berberine, ginkgolide B, baicalin. (**G**–**I**): ow: naringin, fucoidan (a representative sulfated fucose unit, reflecting its polysaccharide nature), astaxanthin. These compounds represent diverse chemical classes and exhibit antioxidant, anti-inflammatory, and anti-apoptotic activities relevant to therapeutic neuroprotection in brain I/R injury.

Numerous natural compounds have shown neuroprotective efficacy when administered before and/or after cerebral I/R injury. However, natural compounds administered after the onset of I/R injury for therapeutic effects or efficacy are limited. This section reviews nine candidates (Table 3 and Figure 3) with therapeutic potential in post-ischemic treatment based on their pharmacological actions, experimental support, and biological relevance (Table 2). Notably, emerging studies have employed advanced in vivo models, such as transient MCAO in rodents [71,72] and embolic stroke models in large animals [73,74], to elucidate these compounds’ efficacy when they are administered after I/R injury. The doses of nine natural compounds used in these studies vary depending on the experimental model, administration route, and compound-specific pharmacokinetics, which is an inherent aspect of preclinical research. Such variability should be taken into account when interpreting therapeutic efficacy. Furthermore, pharmacokinetic profiles, routes of administration, and optimal therapeutic windows are increasingly being investigated, providing essential insights for clinical translation [72,75,76,77,78].

### 3.1. Resveratrol

Resveratrol (C_14_H_12_O_3_) (Figure 3A) is a natural stilbene polyphenol primarily found in grapes, red wine, and berries. It has a molecular weight of 228.24 g/mol and exhibits lipophilic properties, allowing moderate BBB penetration. Its structure comprises two aromatic rings linked by a double bond, which contributes to its antioxidant potential. Biologically, resveratrol scavenges free radicals, modulates signaling pathways such as SIRT1 and Nrf2, and inhibits inflammatory mediators like NF-*κ*B [79,80]. In addition, resveratrol exhibits strong antioxidant, anti-inflammatory, and anti-apoptotic properties [79].

Recent rodent (rat and mouse) studies using MCAO models show that post-treatment with resveratrol (25–50 mg/kg) reduces infarct volume and preserves neurological function, in part by upregulating SIRT1 and Nrf2 [79,81,82]. In hypoxic ischemic injury, SIRT1 activation promotes mitochondrial biogenesis and autophagy, enhancing neuronal resilience to oxidative stress [83]. In addition, post-I/R treatment with resveratrol (100 mg/kg, i.p.) in a rat model of hypoxia-ischemia has been shown to reduce infarct volume, which is related to the inhibition of inflammation and apoptosis [84]. Moreover, in rat MCAO models, post-ischemic administration of resveratrol improved neurological and motor function scores via activation of the SIRT1 pathway [85].

Despite promising neuroprotective effects, resveratrol has poor systemic bioavailability due to extensive first-pass metabolism. In humans, although approximately 70% is absorbed, less than 1% remains unmetabolized in plasma [86]. In rats, the bioavailability of metabolized forms is estimated at around 20% [87].

### 3.2. Curcumin

Curcumin (C_21_H_20_O_6_) (Figure 3B) is a yellow-orange diarylheptanoid polyphenol derived from the rhizome of *Curcuma longa* (turmeric). It has a molecular weight of 368.39 g/mol and consists of two ferulic acid moieties connected by a seven-carbon linker. Despite its poor solubility and bioavailability, curcumin exerts broad pharmacological effects, including antioxidant, anti-inflammatory, and anti-apoptotic actions [88].

Post-ischemic curcumin administration (150 mg/kg) in a mouse MCAO model has been shown to improve sensorimotor function and to reduce infarct volume and inflammatory cytokine release through inhibition of the NF-*κ*B pathway and downregulation of the NOD-, LRR- and pyrin domain-containing protein 3 (NLRP3) inflammasome [5]. In a rat model of MCAO, induction of NLRP3 inflammasome in neurons drives neuroinflammation, and early blockade of NLRP3—achieved through curcumin administration—protects against I/R injury by mitigating inflammation [89]. In addition, curcumin (150 mg/kg) administered after MCAO in mice reduces brain damage and promotes motor function recovery by shifting microglia toward an anti-inflammatory M2 phenotype [90].

However, curcumin exhibits extremely low oral bioavailability due to poor solubility, limited intestinal permeability, and rapid systemic elimination. In humans, its bioavailability is estimated to be less than 1% [91].

### 3.3. Quercetin

Quercetin (C_15_H_10_O_7_) (Figure 3C) is a naturally occurring flavonol present in onions, apples, and berries. With a molecular weight of 302.24 g/mol, it features a typical flavonoid structure including two aromatic rings and a heterocyclic oxygen-containing ring. Quercetin exhibits potent antioxidant activity by scavenging ROS and chelating metal ions [92,93]. In addition, it is suggested that its neuroprotection is potentiated by its synergistic use with zinc or iron chelators [93].

In a rat model of cerebral I/R, quercetin delivered after I/R injury via i.p. injection (30–60 mg/kg) enhances neuronal survival by promoting M2 polarization of microglia/macrophages and modulating the phosphatidylinositol 3-kinase (PI3K)/protein kinase B (Akt)/NF-*κ*B axis [12,93]. In addition, quercetin (50 mg/kg) intraperitoneally administered at the onset of reperfusion in a rat model of MCAO reduces cerebral infarct volume, neurological deficit, BBB permeability, and ROS generation via Sirt1/Nrf2/HO-1 signaling pathway [94]. Moreover, post-ischemic quercetin treatment (5–10 mg/kg) in a rat model of MCAO improved motor function, reduced brain edema, preserved CA1 neurons, and modulated cytokines by increasing IL-10 and decreasing IL-1*β* [95].

Despite its promising pharmacological effects, quercetin has low water solubility and undergoes extensive phase II metabolism, primarily glucuronidation and sulfation, which limits its systemic availability. In a pig model, its oral bioavailability was reported to be less than 0.5% [96]

### 3.4. Berberine

Berberine (C_20_H_18_NO_4_^+^) (Figure 3D) is a quaternary ammonium isoquinoline alkaloid found in *Berberis* species. It has a molecular weight of 336.36 g/mol and is characterized by a planar tetracyclic structure that facilitates DNA intercalation. Biochemically, berberine exhibits antimicrobial, anti-inflammatory, and neuroprotective effects. In brain I/R injury, it reduces ER stress and apoptosis by modulating the Canopy FGF signaling regulator 2 (CNPY2) pathway and preserving mitochondrial integrity [97,98,99].

In a study using a rat MCAO model, fluorescent-labeled berberine analogs were administered after I/R injury to evaluate whether berberine preferentially accumulates in the ischemic brain regions. This study demonstrates selective accumulation of berberine in the ischemic penumbra zones, suggesting its potential for targeted post-I/R neuroprotection [98]. Berberine, at doses of 10–50 mg/kg after I/R injury in rodent models of brain I/R injury, protects neurons by reducing endoplasmic reticulum stress, downregulating the CNPY2 pathway, and enhancing mitochondrial function [97,99]. In addition, post-ischemic berberine administration (40 mg/kg) improved motor function and reduced infarct volume in rat focal cerebral ischemia, accompanied by decreased TNF-*α* and IL-1*β* expression and an increase in IL-10 levels [100].

However, berberine exhibits limited oral absorption due to P-glycoprotein (P-gp) efflux, low intestinal permeability, and extensive first-pass hepatic metabolism. As a result, its oral bioavailability in rats was reported to be less than 0.4%, reflecting substantial intestinal and hepatic elimination [101].

### 3.5. Ginkgolide B

Ginkgolide B (C_20_H_24_O_10_) (Figure 3E) is a diterpene lactone isolated from *Ginkgo biloba* leaves. It has a molecular weight of 424.4 g/mol and a complex cage-like structure rich in hydroxyl groups. This compound is a specific antagonist of platelet-activating factor (PAF), which plays a role in inflammation and vascular permeability [102], and displays anti-atherosclerotic effects [103].

Ginkgolide B, typically administered orally or i.p. at 20 mg/kg in rat MCAO models, enhances post-stroke neurogenesis and neuroplasticity by inhibiting inflammatory cascades [104]. In a rat MCAO model, post-ischemic administration of Ginkgolide B also improves neurological function, reduces infarct volume and brain edema, and attenuates BBB disruption [105]. It acts via PAF antagonism and cyclic AMP response element-binding protein (CREB)-brain-derived neurotrophic factor (BDNF) signaling enhancement [103,104].

Despite its lipophilic nature, ginkgolide B demonstrates limited BBB penetration. However, it is well absorbed following oral administration, with a reported Tmax of approximately 1.5–2 h and a high oral bioavailability of about 88% in humans [106].

### 3.6. Baicalin

Baicalin (C_21_H_18_O_11_) (Figure 3F) is a flavone glycoside extracted from *Scutellaria baicalensis*. It has a molecular weight of 446.37 g/mol and comprises a baicalein core conjugated to a glucuronic acid moiety. Baicalin displays anti-inflammatory and antioxidant activity by modulating cytokines and the expression of apoptosis-related proteins [7,107].

Baicalin, administered at 50–100 mg/kg in rat MCAO models, suppresses NF-*κ*B and cytokines including IL-1*β* and TNF-*α*, suggesting that baicalin upregulates autophagy-related proteins and stabilizes mitochondria [7,107]. In addition, post-ischemic administration of baicalin in a rat MCAO model reduces neurological deficit scores, decreases infarct volume, and lowers NF-*κ*B p65 expression, demonstrating improved motor function and neuroprotection [108].

However, baicalin exhibits poor intestinal permeability and must first be hydrolyzed to baicalein by gut microbiota before absorption. As a result, its oral bioavailability in rats is relatively low, approximately 2.2% [109].

### 3.7. Naringin

Naringin (C_27_H_32_O_14_) (Figure 3G) is a flavanone glycoside abundant in citrus fruits, particularly grapefruit. With a molecular weight of 580.53 g/mol, its structure includes naringenin linked to a neohesperidose sugar. Naringin possesses antioxidant, anti-apoptotic, and neurotrophic properties [110].

In a study using primary cortical neurons from neonatal rats to investigate the neuroprotective properties of naringin, particularly its effects on oxidative stress, administration of naringin shows that naringin’s flavonoid core contributes to scavenging ROS, supporting its antioxidative and neuroprotective roles [110]. Treatment after I/R injury with naringin (100 mg/kg) in a rat MCAO model enhances BDNF levels and neurogenesis, especially in the dentate gyrus, improving spatial memory post-I/R [111]. Moreover, post-ischemic naringin supplementation (100 mg/kg) significantly improved motor function and enhanced neurogenesis in the frontal cortex and hippocampus of ovariectomized female rats after cerebral I/R [112].

Naringin is hydrolyzed to its active metabolite naringenin by gut microbiota and undergoes enterohepatic recirculation, which influences its systemic exposure. Its oral bioavailability has been reported to be approximately 34.4% in dogs and 44.1% in rats, indicating moderate absorption across species [113].

### 3.8. Fucoidan

Fucoidan (Figure 3H, a representative sulfated fucose unit, reflecting its polysaccharide nature) is a heterogeneous sulfated polysaccharide primarily composed of L-fucose and sulfate ester groups. Its chemical structure and molecular weight vary depending on the seaweed source. Fucoidan is known for its antioxidant, anti-inflammatory, and anticoagulant effects [114].

In brain I/R injury, fucoidan treatment (50 mg/kg) during the I/R period weakens inflammatory pathways, oxidative stress, and learning dysfunctions caused by cerebral I/R, showing that strong effects are observed during the simultaneous administration of fucoidan and ruthenium red (a potent inhibitor of intracellular calcium release by ryanodine receptor) [66]. This combination synergistically improves antioxidant and anti-inflammatory efficacy, leading to better neurological outcomes in a rat model of cerebral I/R injury [114]. These findings suggest that the strong inhibition of ryanodine receptor–mediated intracellular calcium release plays a crucial role in enhancing fucoidan’s therapeutic effects.

Fucoidan shows neuroprotective effects but has limited pharmacokinetics due to poor intestinal permeability as a high-molecular-weight sulfated polysaccharide. However, reducing its molecular weight improves bioavailability, with low molecular weight fucoidan exhibiting about 28.3% oral bioavailability in rats, indicating that structural modification can enhance its therapeutic potential [115].

### 3.9. Astaxanthin

Astaxanthin (C_40_H_52_O_4_) (Figure 3I) is a red-orange xanthophyll carotenoid found in microalgae and seafood like salmon and shrimp. It has a molecular weight of 596.84 g/mol and a lipid-soluble structure consisting of conjugated double bonds and terminal rings, which confer antioxidant, anti-inflammatory, and anti-apoptotic properties [116].

In a rat model of cerebral I/R injury, astaxanthin treated at 25–50 mg/kg during cerebral I/R reduces stroke volume, neurological deficits, and apoptosis markers [16]. Its action involves inhibition of lipid peroxidation and mitochondrial protection, restoring caspase-3 levels and oxidant balance [16,116]. Moreover, post-ischemic astaxanthin administration (30 mg/kg) confers neuroprotection in global ischemia, likely via antioxidant mechanisms involving upregulation of HO-1 and heat shock protein (Hsp) 70 [117].

Astaxanthin has strong neuroprotective effects but low oral bioavailability due to its lipophilicity. Its absorption improves with fat-rich meals, with standard astaxanthin showing about 10% bioavailability in humans, while lipid-based formulations increase this to 17–37%, highlighting the benefit of advanced delivery systems [118].

**Table 3:** Summary of natural compounds, their therapeutic properties in brain I/R injury, and their pharmacokinetic properties and bioavailability.

Compound	Formula	Key Functions	Post-I/R Benefits	Key Pharmacokinetic Features	Oral Absorbed Bioavailability (%)	Representative References
Resveratrol	C_14_H_12_O_3_	Antioxidant, anti-inflammatory, SIRT1/Nrf2 activator	Reduces infarct size, suppresses oxidative damage (30–50 mg/kg, mouse MCAO model)	Rapid absorption; however, extensive first-pass metabolism (glucuronidation/sulfation) reduces systemic bioavailability	<1% (unmetabolized), absorbed ~70% (human) ~20% (metabolized form) (rat)	[79,80,81,82,83,84,86,87]
Curcumin	C_21_H_20_O_6_	NF-*κ*B/NLRP3 inhibition, antioxidant	Improve neurological outcomes post-I/R (50–100 mg/kg, rat 150 mg/kg, mouse MCAO model)	Poor solubility and permeability; undergoes rapid conjugation and systemic elimination	<1% (human)	[5,88,89,91]
Quercetin	C_15_H_10_O_7_	PI3K/Akt/NF-*κ*B modulation, promotes M2 microglia	Improves neuronal survival, reduces inflammation (30–60 mg/kg, rat MCAO model)	Low solubility; undergoes extensive phase II metabolism (glucuronidation/sulfation)	<0.5% (pig)	[12,93,94,96]
Berberine	C_20_H_18_NO_4_^+^	Reduces ER stress, mitochondrial protection	Attenuates apoptosis and ER stress post-I/R (10–50 mg/kg, rat MCAO model)	Limited absorption due to P-gp efflux, low permeability, and hepatic first-pass metabolism	<0.4% (rat)	[97,98,99,101]
Ginkgolide B	C_20_H_24_O_10_	PAF antagonist, promotes neurogenesis	Enhance recovery, supports plasticity (20 mg/kg, rat MCAO model)	Well absorbed (Tmax ~1.5–2 h); limited BBB penetration despite lipophilicity	~88% (human)	[102,103,104,106]
Baicalin	C_21_H_18_O_11_	Anti-inflammatory, modulates Bcl-2/Bax, cytokine suppression	Suppresses IL-1*β*, TNF-*α*, reduces infarct size (50–100 mg/kg, rat MCAO model)	Poor permeability; hydrolyzed to baicalein in gut before limited absorption	~2.2% (rat)	[7,109]
Naringin	C_27_H_32_O_14_	BDNF upregulation, supports neurogenesis	Mitigates cognitive deficits post-I/R (100 mg/kg, rat MCAO model)	Hydrolyzed to naringenin by gut flora; undergoes enterohepatic recirculation	~34.4% (dog) ~44.1% (rat)	[110,111,113]
Fucoidan	Variable	Reduces ROS/inflammation, protects BBB	Improves oxidative and inflammatory status (50 mg/kg, rat 4VO model)	Bioavailability increases with lower molecular weight; polysaccharide with poor permeability	~28.3% (Low Molecular Weight Fucoidan) (rat)	[66,114,115]
Astaxanthin	C_40_H_52_O_4_	Antioxidant, stabilizes mitochondria, reduces lipid peroxidation	Reduces stroke volume, restores oxidant/caspase levels (25–50 mg/kg, rat MCAO model)	Lipophilic; bioavailability increases with fat-rich meals	~10% (standard form), ~17–37% (lipid-based formulations) (human)	[16,116,118]

## 4. Mechanisms of Therapeutic Neuroprotection of the Compounds in Brain I/R Injury

**Figure 4:**
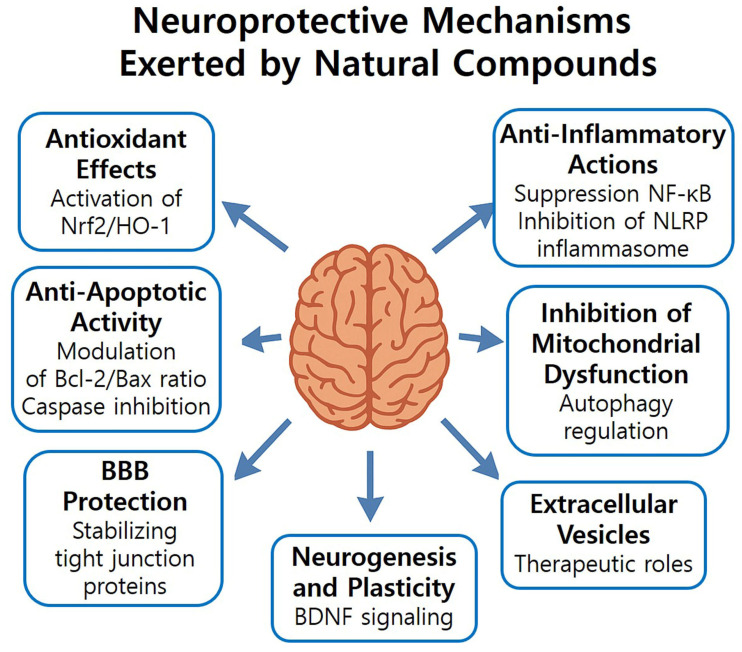
Schematic illustration of the neuroprotective mechanisms exerted by natural compounds following brain I/R injury. These compounds act via multiple pathways, including: activation of the Nrf2/HO-1 antioxidant system; suppression of NF-*κ*B and NLRP3 inflammasome for anti-inflammatory effects; modulation of the Bcl-2/Bax ratio and caspase inhibition for anti-apoptotic effects; protection of the BBB by stabilizing tight junction proteins; promotion of neurogenesis and synaptic plasticity through BDNF signaling; inhibition of mitochondrial dysfunction and autophagy regulation; and therapeutic roles of extracellular vesicles in reducing inflammation and promoting neurogenesis.

Natural compounds exhibit multifaceted therapeutic neuroprotective effects by targeting multiple interconnected pathophysiological mechanisms triggered by brain I/R injury. These mechanisms include oxidative stress, inflammation, apoptosis, BBB disruption, impaired neurogenesis, inhibition of mitochondrial dysfunction, and extracellular vesicle-mediated signaling (Figure 4).

### 4.1. Antioxidant Effects

I/R injury in the brain results in excessive production of ROS, including O_2_^−^, H_2_O_2_, and •OH, which overwhelm endogenous antioxidant defenses and contribute to lipid peroxidation, protein carbonylation, and DNA fragmentation. These oxidative insults ultimately lead to neuronal apoptosis, BBB disruption, and neuroinflammation [119,120]. Natural antioxidant compounds such as curcumin [5], resveratrol [79], and quercetin [121] exert potent neuroprotective effects by modulating cellular redox homeostasis, particularly through activation of the Nrf2/HO-1 signaling pathway. Nrf2 is a key transcription factor that regulates antioxidant response elements in the genome. Under basal conditions, Nrf2 is sequestered in the cytoplasm by Kelch-like ECH-associated protein 1 (Keap1) and targeted for ubiquitin-mediated degradation. However, in the presence of oxidative stress or upon stimulation by polyphenolic compounds, Nrf2 dissociates from Keap1, translocates to the nucleus, and promotes the transcription of antioxidant genes, including HO-1, SOD1/2, glutathione peroxidase (GPx), catalase, and NAD(P)H quinone oxidoreductase 1 (NQO1) [5,79,121].

Curcumin not only activates Nrf2 but also concurrently suppresses the NF-*κ*B pathway and NLRP3 inflammasome, thereby inhibiting microglia/macrophage pyroptosis. This dual modulation reduces oxidative stress and neuroinflammation while protecting white matter integrity in rodent models of ischemic stroke [5]. Curcumin’s ability to cross the BBB, although limited in raw form, is significantly enhanced with nanoparticle or lipid-based delivery systems. Resveratrol has been shown to promote Nrf2 nuclear translocation and HO-1 expression, thereby enhancing endogenous antioxidant capacity and attenuating oxidative neuronal damage after ischemic [79]. In addition to its antioxidative effects, resveratrol also modulates mitochondrial biogenesis via SIRT1 and peroxisome proliferator-activated receptor-gamma coactivator-1 *α* (PGC-1*α*) signaling, which further supports cellular energy homeostasis under hypoxic conditions. Quercetin not only activates the Nrf2/HO-1 axis but also inhibits ferroptosis, a form of iron-dependent cell death triggered by lipid peroxidation. In rodent models of cerebral I/R injury, quercetin treatment upregulated HO-1 expression and suppressed ferroptosis markers such as acyl-CoA synthetase long chain family member 4 (ACSL4) and 4-hydroxynonenal (4-HNE), thereby reducing infarct volume and improving neurological outcomes [121]. Quercetin also stabilizes the mitochondrial membrane and maintains glutathione (GSH) levels, enhancing overall antioxidant resilience. Collectively, these compounds converge on Nrf2-mediated cytoprotection and display distinct secondary targets, enabling complementary neuroprotection. For example, curcumin is more effective at modulating inflammatory cascades, while quercetin is particularly potent against iron-catalyzed lipid peroxidation. These mechanistic differences underscore the potential for combination therapy tailored to the injury phase—acute, subacute, or chronic.

### 4.2. Anti-Inflammatory Actions

Neuroinflammation is a critical component of secondary injury following cerebral I/R and is characterized by the activation of microglia and astrocytes, as well as the upregulation of pro-inflammatory mediators [7]. Natural compounds such as curcumin [5], baicalin [122,123], and berberine [124,125] have been extensively studied for their anti-inflammatory effects in ischemic models. These compounds exert their protective actions by suppressing the NF-*κ*B pathway, a central regulator of inflammation that promotes transcription of cytokines like TNF-*α*, IL-1*β*, and IL-6 [5,7].

Curcumin inhibits NF-*κ*B translocation to the nucleus and blocks its transcriptional activity, which leads to reduced expression of pro-inflammatory cytokines. Furthermore, curcumin significantly downregulates NLRP3 inflammasome components, such as NLRP3 and caspase-1, thereby attenuating the process of pyroptosis, a form of inflammatory cell death common in I/R lesions [5]. Baicalin also exhibits strong anti-inflammatory effects by reducing the activation of NF-*κ*B and decreasing cytokine release in ischemic models. It further modulates mitogen-activated protein kinase (MAPK) signaling, another pathway associated with inflammatory gene expression, contributing to reduced leukocyte infiltration and microglial activation [122,123]. Berberine, an isoquinoline alkaloid, not only downregulates NF-*κ*B and NLRP3 inflammasome activity but also impacts TLR4/MyD88 signaling, which initiates the inflammatory cascade in response to ischemic stress. Through these multifaceted mechanisms, berberine reduces the release of inflammatory mediators and preserves neuronal integrity in the ischemic penumbra [124,125]. Collectively, these compounds mitigate the amplification of post-ischemic inflammation, decrease BBB leakage caused by cytokine-mediated endothelial damage, and ultimately promote a neuroprotective microenvironment conducive to tissue survival and recovery.

### 4.3. Anti-Apoptotic Activity

Apoptosis, or programmed cell death, is a hallmark of neuronal loss in I/R injury, particularly within vulnerable brain regions such as the hippocampal CA1 area and cortical penumbra. Natural compounds, including quercetin, berberine, and astaxanthin, have shown potent anti-apoptotic properties by modulating critical components of the intrinsic (mitochondrial) apoptotic pathway. One of the central molecular mechanisms involves the regulation of the Bcl-2 family proteins, which include anti-apoptotic Bcl-2 and pro-apoptotic Bax. An elevated Bcl-2/Bax ratio stabilizes the mitochondrial membrane potential, preventing cytochrome c release and the subsequent activation of caspase-9 and caspase-3, the executioner caspase responsible for DNA fragmentation and cell dismantling [12,99].

Quercetin enhances neuronal survival after I/R by increasing the Bcl-2/Bax ratio and suppressing cleaved caspase-3 activity. Moreover, it promotes PI3K/Akt pathway activation, which further supports anti-apoptotic signaling and modulates inflammatory gene expression via downstream inhibition of NF-*κ*B [12]. This dual anti-apoptotic and anti-inflammatory mechanism makes quercetin an effective candidate for neuroprotection in both acute and subacute I/R phases. Berberine confers its anti-apoptotic benefit through inhibition of CNPY2 signaling, a pathway that links endoplasmic reticulum stress to mitochondrial apoptotic cascades. This regulatory effect reduces neuronal apoptosis in MCAO models, as evidenced by decreased TUNEL-positive cells and caspase-3 immunoreactivity [99]. Astaxanthin has been reported to exert dose-dependent protection against neuronal apoptosis by directly targeting mitochondrial oxidative stress and membrane destabilization. It decreases the Bax/Bcl-2 ratio and caspase-3 activity while restoring mitochondrial integrity in ischemic models [16]. Additionally, astaxanthin’s antioxidant capacity synergistically enhances its anti-apoptotic effect by mitigating ROS-induced mitochondrial dysfunction. Taken together, these compounds exert their neuroprotective actions by blocking key apoptotic checkpoints, particularly within the intrinsic pathway, and by preserving mitochondrial homeostasis. Their effectiveness suggests potential for adjunctive or combinatory approaches aimed at limiting delayed neuronal death in ischemic stroke.

### 4.4. BBB Protection

The BBB is a specialized structure composed of endothelial cells, pericytes, astrocyte endfeet, and basal lamina. It plays a pivotal role in maintaining cerebral homeostasis by regulating molecular trafficking between the systemic circulation and brain parenchyma. I/R injury disrupts this tightly controlled environment, primarily through oxidative stress, inflammatory cytokines, and MMP activation, which degrade tight junction proteins such as occludin and claudin-5. This results in increased vascular permeability, leukocyte infiltration, and vasogenic edema, contributing to secondary brain injury [126,127,128].

Curcumin has been shown to protect BBB integrity through multiple mechanisms. In rodent models of focal and global cerebral I/R, curcumin administration preserves the expression of occludin and claudin-5, mitigates BBB leakage (as evidenced by Evans blue extravasation), and decreases brain water content, indicating reduced edema [129,130]. Fucoidan also demonstrates BBB-protective effects through anti-inflammatory and antioxidant properties. It inhibits endothelial activation and prevents the upregulation of cell adhesion molecules (e.g., P-selectin and intercellular adhesion molecule 1 (ICAM-1)), thereby suppressing leukocyte adhesion and transmigration across the vascular wall [66]. This ultimately attenuates microvascular injury and tight junction disruption. Together, these compounds help to preserve structural and functional components of the BBB, limiting immune cell infiltration, cerebral edema, and neuronal damage during the reperfusion phase. Their ability to target both molecular and cellular regulators of BBB breakdown makes them promising candidates for adjunctive neurovascular protection in ischemic stroke.

### 4.5. Neurogenesis and Plasticity

I/R injury not only causes acute neuronal death but also impairs neurogenesis and synaptic plasticity. Post-ischemic neurogenesis is often disrupted due to oxidative stress, inflammation, and microenvironmental changes that impair the proliferation and differentiation of neural stem/progenitor cells. However, certain molecular signals—especially BDNF—are known to facilitate neurogenic recovery and plastic remodeling. Experimental studies have shown that BDNF expression is upregulated during the post-ischemic repair phase and contributes to the restoration of synaptic integrity through tropomyosin receptor kinase B (TrkB)-mediated signaling pathways. For instance, increased BDNF and doublecortin-positive cells in the dentate gyrus have been observed in rodent models of global cerebral I/R, indicating enhanced neurogenesis and neuronal integration [131].

Natural compounds like naringin [111] and ginkgolide B [104] contribute significantly to post-ischemic brain repair through modulation of neurogenesis and synaptic plasticity. These compounds enhance the expression of BDNF, a critical regulator of neuronal survival, differentiation, and synaptic remodeling, particularly in the hippocampus, which is vulnerable to I/R injury. In rodent models of transient global cerebral ischemia, naringin treatment has been shown to elevate hippocampal BDNF expression, which correlates with increased neurogenesis in the dentate gyrus and improved spatial memory performance in behavioral tests [111]. These findings indicate that both compounds exert neurorestorative effects by enhancing endogenous neuroplastic mechanisms. Importantly, their combined use with antioxidants or anti-inflammatory agents may synergistically optimize recovery outcomes by concurrently protecting neurons and promoting their functional reintegration.

### 4.6. Inhibition of Mitochondrial Dysfunction

In the context of I/R injury, mitochondria are central mediators of neuronal survival or death. Mitochondrial impairment promotes oxidative stress, calcium dysregulation, and apoptosis, which aggravate brain damage. Recent studies emphasize that targeting mitochondrial bioenergetics and quality control pathways may offer therapeutic benefit. For instance, it is identified that the activation of mitochondrial complex I and downstream nitric oxide-mediated signaling contributes to delayed cell death after brain ischemia, but pharmacological inhibition of mitochondrial dysfunction reduces infarct size and neurodegeneration [47,132]. Moreover, it is demonstrated that regulation of mitochondrial fission/fusion dynamics and mitophagy plays a critical role in maintaining mitochondrial homeostasis after cerebral I/R. The study shows that therapeutic interventions modulating mitochondrial membrane potential and activating protective autophagy improve neuronal viability [47].

### 4.7. Extracellular Vesicles as Neuroprotective Mediators

Extracellular vesicles (EVs) released from damaged or stressed neurons and glial cells carry bioactive molecules such as microRNAs, inflammatory mediators, and mitochondrial fragments. Recently, the dual role of EVs in cerebral I/R injury has been emphasized. On one hand, EVs propagate pro-inflammatory and apoptotic signals from damaged neural cells. On the other hand, therapeutic EVs—especially those derived from mesenchymal stem cells—demonstrate the ability to modulate immune responses, promote angiogenesis, and facilitate neurogenesis [133,134]. These findings suggest that engineered EVs represent a novel therapeutic modality for enhancing brain repair and functional recovery after I/R injury.

## 5. Mechanistic Comparison of the Compounds and Insights

Although both quercetin and curcumin activate the Nrf2 signaling pathway, their downstream modulatory effects diverge in clinically meaningful ways. Quercetin promotes anti-inflammatory microglial M2 polarization [135], while curcumin inhibits pyroptosis by downregulating NLRP3 inflammasome activation, a key driver of post-ischemic inflammation [136]. These complementary effects suggest potential for phase-specific interventions—curcumin being particularly beneficial in the acute pro-inflammatory phase, while quercetin may support subacute neurorepair via immunomodulation. Similarly, berberine and baicalin share the ability to modulate apoptosis-related proteins such as Bcl-2 and Bax, yet act through distinct upstream mechanisms. Berberine primarily attenuates ER stress and enhances mitochondrial integrity [137], whereas baicalin reduces pro-inflammatory cytokine release and may also activate autophagy pathways [15]. These mechanistic differences broaden the therapeutic scope of these compounds and indicate their potential for synergistic combination therapy targeting multiple injury cascades. When combining natural compounds, both synergistic and antagonistic interactions may occur [138], underscoring the importance of dose–response assessment and interaction profiling in the design of combination therapies. Regarding translational applicability, resveratrol and curcumin have both demonstrated efficacy across species, including in rodent models and human studies, and recent advances in nanocarrier systems have further enhanced the brain-targeting capacity of natural compounds [139]. Resveratrol shows low oral bioavailability in both humans and animals, though it is metabolized more rapidly in rodents, which may lead to underestimation of its efficacy in preclinical models [140,141]; however, ApoE-modified resveratrol nanoliposomes have been shown to enhance BBB transport and improve cognitive outcomes in Alzheimer’s disease models [142]. Curcumin, despite similar bioavailability limitations, has shown enhanced therapeutic index via nanoparticle formulations, particularly in rodent studies [143], with erythrocyte membrane-coated nanoparticles improving BBB penetration and providing therapeutic benefits in neurodegenerative disease models [144]. Similarly, baicalin delivery was significantly enhanced using borneol-liposomes and macrophage membrane-coated liposomes, facilitating better brain accumulation in cerebral ischemia models [145,146]. These findings reinforce the importance of delivery system optimization and pharmacokinetic tailoring to bridge preclinical and clinical efficacy. In brief, while many compounds share overlapping targets such as Nrf2, NF-*κ*B, or Bcl-2, each possesses unique mechanistic attributes and optimal time windows of intervention. Recognizing these distinctions and strategically combining agents with complementary actions may significantly enhance post-ischemic recovery. This mechanistic diversity, rather than being a limitation, represents a valuable asset in designing multifaceted, stage-specific therapeutic strategies.

## 6. Challenges and Opportunities in Translating Natural Compounds for Brain I/R Therapy

**Figure 5:**
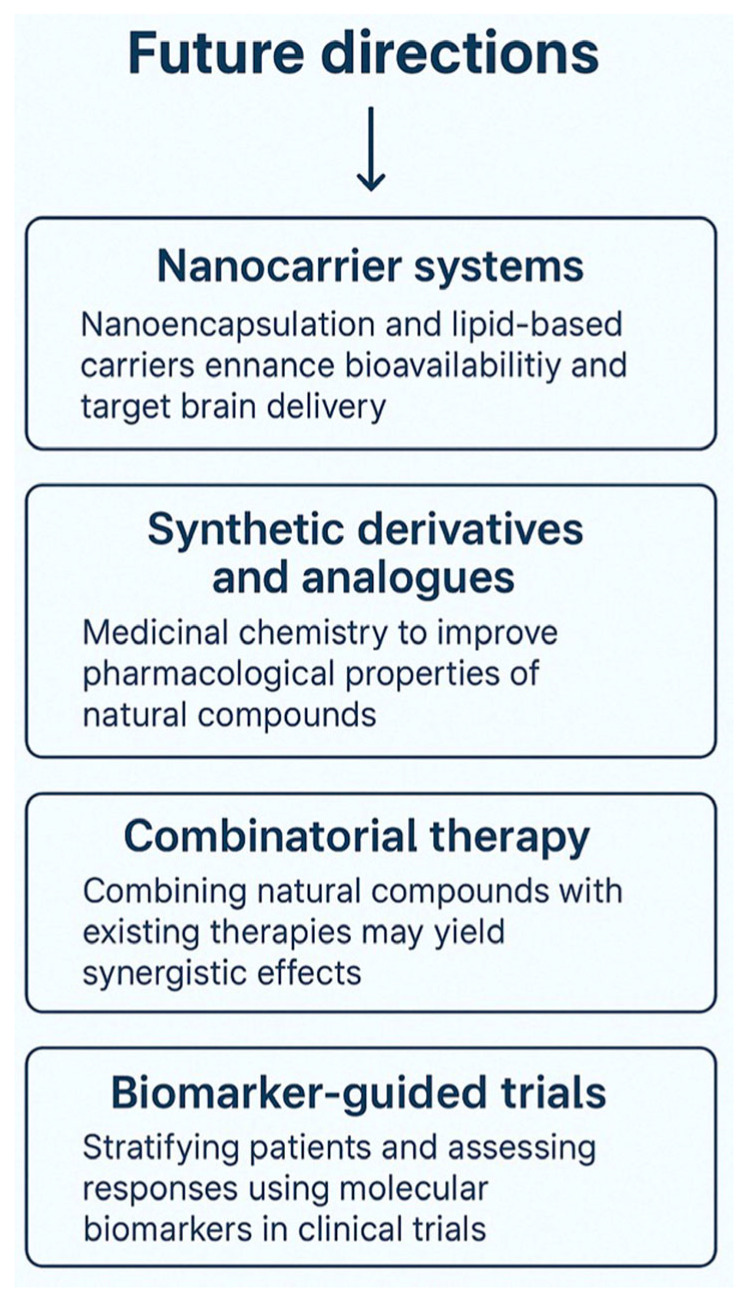
Schematic overview of future strategies to enhance the therapeutic application of natural compounds for brain I/R injury. This diagram outlines four major future directions aimed at overcoming current limitations in the clinical translation of natural compounds. These strategies, supported by formulation science and precision medicine, are critical for advancing natural compounds from preclinical promise to clinical impact.

While natural compounds show promising therapeutic effects in preclinical models of brain I/R injury, several translational challenges remain. However, these challenges have spurred substantial innovation, transforming limitations into opportunities for advancement. First, the low oral bioavailability of compounds such as curcumin and resveratrol—due to rapid metabolism and poor solubility—has prompted the development of novel delivery systems to enhance therapeutic exposure in the brain [147,148,149]. Second, although BBB penetration remains a hurdle, innovative strategies such as nanoparticles, peptide shuttles, and intranasal delivery systems are significantly improving brain-targeted delivery [150,151,152]. Third, variability in botanical sources and extraction methods has historically hindered reproducibility; yet, standardization protocols and chemical fingerprinting techniques are now being widely adopted to ensure batch consistency and quality control [153,154]. Fourth, the lack of standardized dosages and pharmacokinetic profiles in experimental models has limited comparability across studies. This issue is being actively addressed through the development of harmonized preclinical guidelines and pharmacokinetic modeling tools [2]. Finally, while clinical trials remain limited, the growing interest in evidence-based phytotherapy and the integration of biomarkers into trial design offer a path forward for validating efficacy and identifying responsive patient populations [155]. To accelerate translation, multiple promising approaches are being investigated (Figure 5). These include: (1) Nanocarrier systems (e.g., liposomes, micelles, PLGA nanoparticles) that protect bioactive molecules and improve BBB transport [156,157]; (2) Synthetic derivatives and analogues created through medicinal chemistry to enhance stability, solubility, and potency [158,159]; (3) Combination therapy using natural compounds alongside thrombolytics or anti-inflammatory agents to amplify neuroprotection [7]; and (4) Biomarker-guided clinical trials, which allow precise patient stratification and real-time assessment of therapeutic responses [160,161]. In brief, the field is moving beyond identifying limitations to building solutions—driven by interdisciplinary collaboration among pharmacologists, neuroscientists, formulation scientists, and clinicians. With continued innovation and strategic investment in translational research, natural compounds hold strong potential as safe, multifaceted therapies for patients suffering from ischemic brain injury.

## 7. Conclusions

This review highlights the growing body of evidence supporting the therapeutic potential of natural compounds in mitigating brain I/R injury. Compounds such as resveratrol, curcumin, quercetin, and others exhibit diverse bioactivities, including antioxidant, anti-inflammatory, anti-apoptotic, and neurogenic effects. These multifaceted actions target the complex molecular cascades of I/R injury, promoting therapeutic neuroprotection and functional recovery in preclinical models. Importantly, many of these compounds act on convergent signaling pathways such as Nrf2, NF-*κ*B, and SIRT1, offering synergistic opportunities when used alone or in combination. Although certain translational barriers remain—such as limited bioavailability, BBB permeability, interspecies differences, variability in clinical protocols, and the lack of toxicity data for high-dose or long-term use, as well as the current lack of robust clinical studies—these are increasingly being addressed through nanotechnology, structural optimization, and biomarker-guided clinical trial design. As discussed, recent advancements in formulation science, delivery systems, and interdisciplinary research are closing the gap between experimental efficacy and clinical applicability. With continued innovation and validation, natural compounds may evolve into low-toxicity, accessible interventions capable of improving neurological outcomes in patients with ischemic stroke and related cerebrovascular disorders.

## Data Availability

Data sharing is not applicable.

## Abbreviations

The following abbreviations are used in this manuscript:

Akt	Protein kinase B
AMPA	*α*-amino-3-hydroxy-5-methyl-4-isoxazolepropionic acid
ATP	Adenosine triphosphate
BBB	Blood-brain barrier
BCCAO	Bilateral common carotid artery occlusion
Bax	Bcl-2-associated X protein
Bcl-2	B-cell leukemia/lymphoma 2
BDNF	Brain-derived neurotrophic factor
CNPY2	Canopy FGF signaling regulator 2
EVs	Extracellular vesicles
HO-1	Heme oxygenase-1
IL	Interleukin
IgG	Immunoglobulin G
I/R	Ischemia-Reperfusion
Keap1	Kelch-like ECH-associated protein 1
MCAO	Middle cerebral artery occlusion
MMPs	Matrix metalloproteinases
mPTP	Mitochondrial permeability transition pore
NF-*κ*B	Nuclear factor kappa B
NLRP3	NOD-, LRR- and pyrin domain-containing protein 3
NMDA	N-methyl-D-aspartate
Nrf2	Nuclear factor erythroid 2-related factor 2
PAF	Platelet-activating factor
PI3K	Phosphatidylinositol 3-kinase
ROS	Reactive oxygen species
SOD	Superoxide dismutase
TIA	Transient ischemic attack
TNF-*α*	Tumor necrosis factor alpha
TUNEL	TdT-mediated dUTP nick end labeling
4VO	four-vessel occlusion
